# A new superinvasive *in vitro* phenotype induced by selection of human breast carcinoma cells with the chemotherapeutic drugs paclitaxel and doxorubicin

**DOI:** 10.1038/sj.bjc.6602221

**Published:** 2004-10-26

**Authors:** S A Glynn, P Gammell, M Heenan, R O'Connor, Y Liang, J Keenan, M Clynes

**Affiliations:** 1 National Institute for Cellular Biotechnology, Dublin City University, Glasnevin, Dublin 9, Ireland

**Keywords:** paclitaxel (taxol), doxorubicin (adriamycin), drug resistance, invasion, motility, adhesion, Rho-GTPases

## Abstract

Doxorubicin- and paclitaxel-selected variants of an *in vitro* invasive clonal population of the human breast cancer cell line, MDA-MB-435S, were established by pulse selection, and exhibited a novel ‘superinvasive’ phenotype. This phenotype is characterised by an ability to relocate to another surface following invasion through matrigel and membrane pores, by decreased adhesion to extracellular matrix proteins and by increased motility. This may represent an *in vitro* model of a step in the metastatic process occurring subsequent to invasion. The paclitaxel-resistant variants, MDA-MB-435S-F/Taxol-10p and MDA-MB-435S-F/Taxol-10p4p were resistant to paclitaxel, vincristine and docetaxel, but not to doxorubicin, carboplatin, etoposide or 5-fluorouracil. The doxorubicin-selected variants MDA-MB-435S-F/Adr-10p and MDA-MB-435S-F/Adr-10p10p, in contrast, exhibited only small increases in resistance to doxorubicin, although they were slightly resistant to VP-16 and docetaxel, and exhibited increased sensitivity to paclitaxel, carboplatin and 5-fluorouracil.

Invasion and metastasis are intimately involved in the pathology of cancer, but the biological and molecular natures of these phenomena remain incompletely understood. A possible association exists between multiple drug resistance and invasive potential in carcinoma cells (Liang *et al*, 2001, 2002).

The majority of previous studies focus on the direct effect of drug exposure on the invasive potential (invasion in the presence of the drug), as opposed to the long-term effects of drug treatment on the invasive phenotype of cell, coupled with emerging drug resistance. While a few reports indicate that paclitaxel exposure increases motility and invasion (Welch *et al*, 1989; Silbergeld *et al*, 1995), others indicate an inhibitory effect on invasion (Westerlund *et al*, 1997; Sgadari *et al*, 2000). Silbergeld *et al* (1995) reported that increasing the paclitaxel concentration caused increased locomotion in glioblastoma cell lines. Welch *et al* (1989) showed that pretreatment of cell lines, with low and high invasive potentials, with vincristine, colcemid, or colchicine (but not paclitaxel), at noncytotoxic levels, resulted in inhibition of invasion. Westerlund *et al* (1997) showed that OVCAR-3 cell attachment, migration and *in vitro* invasion were significantly decreased after paclitaxel treatment. Sgadari *et al* (2000) found that paclitaxel promoted regression of Kaposi's sarcoma (KS) lesions *in vivo* and that it blocked the growth, migration, and invasion of KS cells *in vitro*. Doxorubicin appears to have an inhibitory effect on invasion (Repesh *et al*, 1993; Hikawa *et al*, 2000).

Previous studies in this laboratory showed that selection of RPMI 2650, a noninvasive cell line, with the chemotherapeutic agents paclitaxel and melphalan, resulted in multiple drug-resistant phenotypes and in the case of melphalan but not paclitaxel, a highly invasive phenotype (Liang *et al*, 2001). To further investigate the effects of chemotherapeutic drug selection, in particular doxorubicin and paclitaxel, and the effect of these drugs on adhesive, locomotive and invasive phenotypes, paclitaxel- and doxorubicin-selected variants of the human breast cell line MDA-MB-435S-F were established.

## MATERIALS AND METHODS

### Chemicals

Paclitaxel and VP-16 were obtained from Bristol-Myers Squibb (Dublin, Ireland), doxorubicin and 5-fluorouracil (5-FU) from Farmitalia Carlo Erba (Milton Keynes, UK), docetaxel from Aventis (France) and vincristine from Lederle (Dublin, Ireland). All media and serum used in the maintenance of the cell lines were obtained from Sigma Aldrich (Dublin, Ireland). Unless stated, all other chemicals were obtained from Sigma (Dublin, Ireland).

### Cell lines

The human breast cancer cell line MDA-MB-435S was obtained from the American type culture collection (ATCC) and was maintained in culture at 37°C using RPMI supplemented with 2 mM L-glutamine (Gibco) and 10% foetal calf serum. MDA-MB-435S is a spindle-shaped strain which evolved from the parental line MDA-MB-435 isolated by Cailleau *et al* (1978), derived from the pleural effusion of a 31-year-old female with metastatic, ductal adenocarcinoma of the breast. MDA-MB-435S-F was obtained by clonal dilution from MDA-MB-435S in this laboratory. The MDA-MB-435S-F doxorubicin- or paclitaxel-resistant variants were derived by pulse selection of the parental cells with the IC_90_ value of doxorubicin or paclitaxel. Cells were exposed to the IC_90_ value of 15 ng ml^−1^ paclitaxel for 4 h, once a week for 10 weeks (MDA-MB-435S-F/Taxol-10p). These cells were then exposed to 125 ng ml^−1^ paclitaxel for 4 h, once a week for 4 weeks (MDA-MB-435S-F/Taxol-10p4p). Cells were also exposed to the IC_90_ value of 120 ng ml^−1^ doxorubicin for 4 h, once a week for 10 weeks (MDA-MB-435S-F/Adr-10p). These cells were then exposed to a further 10 pulse with 200 ng ml^−1^ doxorubicin for 4 h, once a week for 10 weeks (MDA-MB-435S-F/Adr-10p10p). Antibiotics were not used in the growth media. All cell lines were free from mycoplasma as tested with the indirect Hoechst DNA staining method.

### Western blotting

Western blotting was performed by the method of Moran *et al* (1992). Cdc-42, Rho A, Rac1, MMP-2, c-Met, IGF-1R Pgp, MRP1, EGFR, HER2, HER3 and HER4 were detected by Western blotting with polyclonal antibodies AB4201, AB19016, monoclonal antibodies MAB3735 (Chemicon, UK), polyclonal antibody sc-28, sc-713 (Santa Cruz Biotechnology, USA), monoclonal antibodies ALX-801-002 and ALX-801-007 (Alexis, UK), monoclonal MS-400-P1, MS-327-P, MS-313-P and polyclonal antibodies RB-284-P (Neomarkers, USA), respectively. Secondary antibodies rabbit anti mouse-HRP (P0260), goat anti-rabbit-HRP (P0448) and rabbit anti-Rat-HRP (P0450) were obtained from Dako (Germany).

### Cytotoxicity assays

Cytotoxicity testing of drugs was measured by colorimetric assays as previously described (Martin and Clynes, 1991, 1993; Duffy *et al*, 1998). Briefly on day 1, cells were seeded at 1 × 10^3^ cells/well in a 96-well plate and allowed to attach overnight in a 5% CO_2_ incubator at 37°C. The appropriate concentrations of drug were added to the plate on day 2, and the assay was terminated on day 7. All assays were performed in triplicate.

### Quantification of doxorubicin accumulation in MDA-MB-435S-F

The level of doxorubicin in DLKP cells was quantified using liquid – liquid extraction and reverse-phase high-performance liquid chromatography (HPLC) analysis as previously described in Duffy *et al* (1998).

### Adhesion assays

Adhesion assays were performed using the method of Torimura *et al* (1999). 24-well plates were coated with 250 *μ*l of 25 *μ*g ml^−1^ collagen type IV, fibronectin and laminin and 250 *μ*l of 1 mg ml^−1^ Matrigel and incubated overnight at 4°C. A volume of 0.5 ml of a sterile 0.1% BSA/PBS solution was dispensed into each well to reduce nonspecific binding. The plates were incubated at 37°C for 20 min and then rinsed twice with PBS. Cells were resuspended in serum-free RPMI medium and plated at a concentration of 2.5 × 10^4^ cells well^−1^ in triplicate and incubated at 37°C for 60 min. Control wells were those that had been coated but contained no cells. After 60 min, the medium was removed from the wells and rinsed gently with PBS. This was then removed and 200 *μ*l of freshly prepared phosphatase substrate (10 mM *p*-nitrophenol phosphate in 0.1 M sodium acetate, 0.1% Triton X-100, pH 5.5) was added to each well. The plates were then incubated in the dark at 37°C for 2 h. The enzymatic reaction was stopped by the addition of 100 *μ*l of 1 N NaOH. 100 *μ*l aliquots were transferred to a 96-well plate and read in an ELISA reader at 405 nm with a reference wavelength of 620 nm.

### Invasion assays and motility assays

Invasion assays were performed by a modification of the method described by Albini *et al* (1991). Matrigel was diluted to 1 mg ml^−1^ in serum-free DMEM medium. A volume of 100 *μ*l of 1 mg ml^−1^ Matrigel was placed into each insert (Falcon) (8.0 *μ*m pore size), which stood in wells of a 24-well plate (Costar). The inserts and the plate were incubated overnight at 4°C. The following day, cells were harvested and suspended in RPMI 1640 containing 10% FCS at a concentration of 1 × 10^6^ cells ml^−1^. The inserts were washed with serum-free RPMI 1640, then 100 *μ*l of the cell suspension was added to each insert and 250 *μ*l of RPMI 1640 containing 10% FCS was added to the insert well. Cells were incubated at 37°C for 48 h. After this time period, the inner side of the insert was wiped with a wet swab to remove the cells, while the outer side of the insert was gently rinsed with PBS and stained with 0.25% crystal violet for 10 min, rinsed again and then allowed to dry. The surface of the 24-well plate was rinsed gently twice with PBS and stained with 0.25% crystal violet for 10 min, rinsed again and then allowed to dry. Cells were quantified by both counting with the aid of a light microscope and eluting the stain with 200 *μ*l 33% glacial acetic acid. Aliquots (100 *μ*l) were transferred to a 96-well plate and read in an ELISA reader at 570 nm with a reference wavelength of 620 nm. The procedure for carrying out motility assays was identical to the procedure used for invasion assays with the exception that the inserts were not coated with Matrigel.

### Gelatin zymography

Zymography was used to assess the level of proteolytic activity of different proteases. Gelatin is a substrate for MMPs, serine and cysteine proteases. The protein concentration of the serum-free medium in which cells were cultured was determined by the Biorad protein assay (Biorad) and 20 *μ*g of supernatant protein was applied to nonreduced sodium dodecyl sulphate – polyacrylamide gel electrophoresis (SDS – PAGE) using a 10% gel containing 0.1% gelatin. After electrophoresis, gels were soaked in 2.5% Triton X-100 at room temperature with gentle shaking for 30 min and incubated in substrate buffer±EDTA (50 mM Tris-HCl, pH 8.0, 5 mM CaCl_2_) at 37°C for 18–24 h. Gels were then stained with 2.5 mg ml^−1^ Coomassie Blue for 2 h and destained in a mixture of acetic acid : isopropanol: distilled water (1 : 3 : 6) until clear bands were visible. Gelatinase activity was determined as distinct, clear bands. Each zymograph was carried out three times.

### Statistical analysis

Analysis of the significance of the difference in the mean IC_50_ value calculated from toxicity assays were performed using a paired Student's *t*-test, which was run on Sigma Plot. A *P*-value >0.05 was deemed not significant. A *P*-value <0.05 was deemed significant. A *P*-value <0.005 was deemed highly significant.

## RESULTS

### Motility and invasion assays

Figure 1

**Figure 1:**
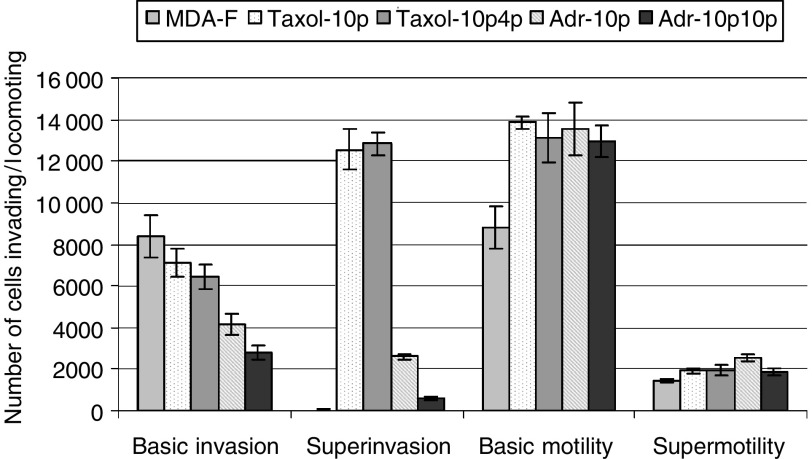
Levels of basic invasion, superinvasion, basic motility and supermotility are displayed for MDA-MB-435S-F, MDA-MB-435S-F/Taxol-10p, MDA-MB-435S-F/Taxol-10p4p, MDA-MB-435S-F/Adr-10p and MDA-MB-435S-F/Adr-10p10p. Results are displayed as the mean number of cells invading or locomoting±s.d. (*n*=3).

describes the invasive and locomotive capabilities of MDA-MB-435S-F. Figure 1 shows that MDA-MB-435S-F is highly invasive, but selection with paclitaxel or doxorubicin results in a more aggressive invasive phenotype characterised by a proportion of the invading cells detaching from the bottom of the insert and reattaching to the bottom of the 24-well plate (superinvasion), which was negligible in MDA-MB-435S-F. This effect was greater for the paclitaxel-selected than doxorubicin-selected variants. *In vitro* invasiveness for the positive control cell line RPMI-2650Ml and negative control cell line RPMI-2650 (Liang *et al*, 2001) gave expected results (results not shown) and the superinvasive phenotype was not observed in the positive control. Motility assays in Figure 1 show that MDA-MB-435S-F is motile. The motility of both the paclitaxel- and the doxorubicin-selected variants were found to have further increased. In contrast to what was observed in invasion assays, following motility assays, a proportion of parental MDA-MB-435S-F cells had detached from the bottom of the insert and reattached to the bottom of the 24-well plate. This effect was also observed in the drug-selected variants.

### Adhesion assays

**Figure 2:**
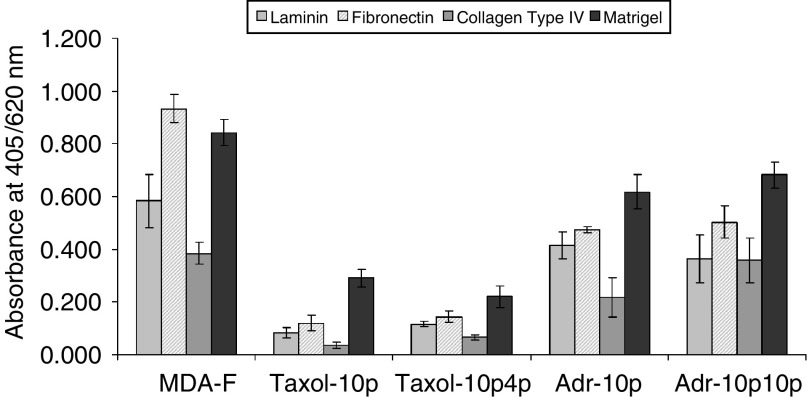
Attachment of the MDA-MB-435S-F cell line and its paclitaxel- and doxorubicin-selected variants to extracellular matrix (ECM) proteins: laminin, fibronectin, collagen type IV, and matrigel. Results are expressed as absorbance at 405 nm with a reference wavelength of 620 nm. The data are shown as mean absorbance±s.d. (*n*=3).

Paclitaxel- or doxorubicin-selection of MDA-MB-435S-F results in decreased adhesion to fibronectin, laminin, collagen type IV and Matrigel (Figure 2

). This decrease is more apparent in paclitaxel-selected cell lines than in doxorubicin-selected cell lines (mirroring the degree of expression of the superinvasive phenotype).

### Cross-resistance profiles

The sensitivity of the paclitaxel-selected variants, and the doxorubicin-selected variants, to a range of chemotherapeutic agents was determined (Tables 1

**Table 1:** Comparison of chemosensitivity in the MDA-MB-435S-F cell line and its paclitaxel selected variants

	**IC50 (ng ml^−1^)**				
**Drug**	**MDA-MB- 435S-F**	**MDA-MB-435S- F/Taxol-10p**	**Fold resistance of MDA-MB-435S- F/Taxol-10p**	**MDA-MB-435S- F/Taxol-10p4p**	**Fold resistance of MDA-MB-435S- F/Taxol-10p4p**
Doxorubicin	271±3	242±21	0.89	456±20**	1.68
Paclitaxel	28.3±1.52	259±3**	9.15	900±40**	31.80
Carboplatin	41 300±1500	54 300±5100*	1.31	33 000±3400	0.79
Docetaxel	3.33±0.37	12.23±0.37**	3.67	62.4±1.3**	18.73
VP-16	1844±77	2225±152	1.20	1740±17	0.94
5-Fluorouracil	7430±680	3500±170**	0.47	6830±600	0.91
Vincristine	12.9±1.3	74.6±0.5**	5.78	176±56**	13.64

a The IC_50_ values are the mean of a minimum of three repeat experiments.

* *P*<0.05, Student's *t*-test.

** *P*<0.005, Student's *t*-test.

and 2

**Table 2:** Comparison of chemosensitivity in the MDA-MB-435S-F cell line and its doxorubicin selected variants

	**IC50 (ng ml^−1^)**				
**Drug**	**MDA-MB- 435S-F**	**MDA-MB-435S- F/Adr-10p**	**Fold resistance of MDA-MB-435S-F/Adr-10p**	**MDA-MB-435S- F/Adr-10p10p**	**Fold resistance of MDA-MB-435S-F/Adr-10p10p**
Doxorubicin	271±3	316±15	1.16	381±11.54**	1.40
Paclitaxel	28.3±1.52	20.3±2.1*	0.71	17.6±0.2*	0.62
Carboplatin	41 300±1500	37 600±1150	0.91	24 600±1520*	0.59
Docetaxel	3.33±0.37	3.76±0.20	1.12	4.76±0.152*	1.42
VP-16	1844±77	2663±148**	1.44	3785±79**	2.05
5-Fluorouracil	7430±680	3360±400*	0.45	3430±50*	0.46
Vincristine	12.9±1.3	6.9±0.69**	0.53	32.1±2.5**	1.61

a The IC_50_ values are the mean of a minimum of three repeat experiments.

* *P*<0.05, Student's *t*-test.

** *P*<0.005, Student's *t*-test.

). The highest levels of resistance in the paclitaxel-selected variants were observed with vincristine, docetaxel and its selecting agent paclitaxel (Table 1). A low-level resistance to carboplatin and a decreased sensitivity to 5-fluorouracil were observed for MDA-MB-435S-F/Taxol-10p, but not in MDA-MB-435S-F/Taxol-10p4p. MDA-MB-435S-F/Taxol-10p4p developed a low-level resistance to doxorubicin. Resistance to VP-16 was unaltered in these variants. The results (Table 2) show that MDA-MB-435S-F failed to develop any significant resistance to doxorubicin by pulse selection, despite the fact that high cell kill was observed during selection with doxorubicin. However MDA-MB-435S-F/Adr-10p was found to be marginally resistant to VP-16 and sensitised to 5-fluorouracil, vincristine and paclitaxel. No significant change in resistance to carboplatin or docetaxel was observed. MDA-MB-435S-F/Adr-10p10p exhibited crossresistance to docetaxel, vincristine, and VP-16, but was sensitised to paclitaxel, carboplatin and 5-fluorouracil.

### Detection of drug efflux proteins Pgp and MRP-1 and results of doxorubicin efflux studies using HPLC analysis and circumvention studies with sulindac and verapamil

Figure 3(a)

**Figure 3:**
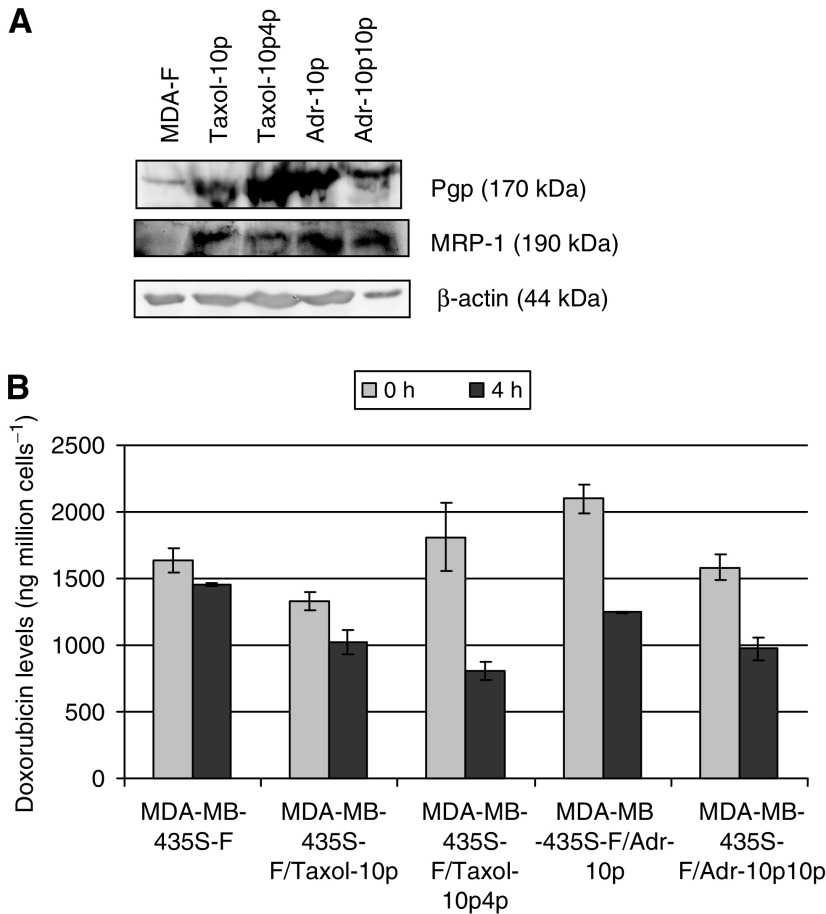
Expression of (**A**) drug efflux associated proteins, Pgp and MRP1, and levels of (**B**) doxorubicin accumulation and efflux (±s.d.; *n*=3) in MDA-MB-435S-F and its doxorubicin- and paclitaxel-selected variants.

shows that all pulse selected MDA-MB-435S-F variants exhibit increased levels of Pgp (p-glycoprotein) and MRP-1 by Western blot. Analysis of doxorubicin uptake and efflux was carried out on MDA-MB-435S-F variants, using quantification by HPLC (Figure 3(b)) and all were found to exhibit an increase (range two- to five-fold; MDA-MB-435S-F/Taxol-10p – 2.1X, MDA-MB-435S-F/Taxol-10p4p – 5.0X, MDA-MB-435S-F/Adr-10p – 3.6X, MDA-MB-435S-F/Adr-10p10p – 3.5X) in doxorubicin *efflux* compared with the parental cell line. Toxicity assays were performed with doxorubicin and paclitaxel, combined with either sulindac (MRP-1 inhibitor) or verapamil (Pgp and MRP-1 inhibitor) (data not shown). Both verapamil and sulindac enhanced doxorubicin toxicity in all cell lines (parental and variants), but verapamil had a greater effect than sulindac on all selected variants. Verapamil (but not sulindac) had an effect on paclitaxel resistance but only in the paclitaxel-selected variants (taxanes are known to be poor substrates for MRP-1). Sulindac had no effect on paclitaxel toxicity in the parental cell line or either of the doxorubicin-selected variants, but note that these were already more sensitive to paclitaxel than the parental line. These results suggested increased activity of at least two efflux pumps as a result of doxorubicin- and paclitaxel-selection. Doxorubicin net *accumulation* in the variants was however variable in comparison to the parental cell line (MDA-MB-435S-F/Taxol-10p – 81.45±5.3%, MDA-MB-435S-F/Taxol-10p4p – 110.78±14.1%, MDA-MB-435S-F/Adr-10p – 128.41±5.3%, MDA-MB-435S-F/Adr-10p10p – 97.06±6.1%). These results suggest that factors other than efflux may also play a role in net drug accumulation in these cell lines.

### Isolation and morphology of populations of differing invasive population

Three populations of cells were isolated from MDA-MB-435S-F and its drug-selected variants. To do this invasion assays were set up and the cells were allowed to invade for 48 h. The three populations were isolated as follows. Firstly, cells which had not invaded (noninvasive, NI), were isolated from the inside of the invasion chamber by pipetting up remaining cells and transferring to a well containing fresh medium. A second population (invasive, I) was isolated from the bottom side of the insert by trypsinisation of cells into a well containing fresh medium. The third population (superinvasive, SI), that is, those cells which had invaded, detached from the bottom of the insert and reattached to the bottom of the 24-well plate, were allowed to grow until they reached suitable confluency levels for further subculturing. No differences were observed in the morphology of the three invasive populations isolated, except for an increased tendency of invasive and superinvasive cells to grow in clumps even at subconfluency.

### Invasiveness of isolated populations

**Figure 4:**
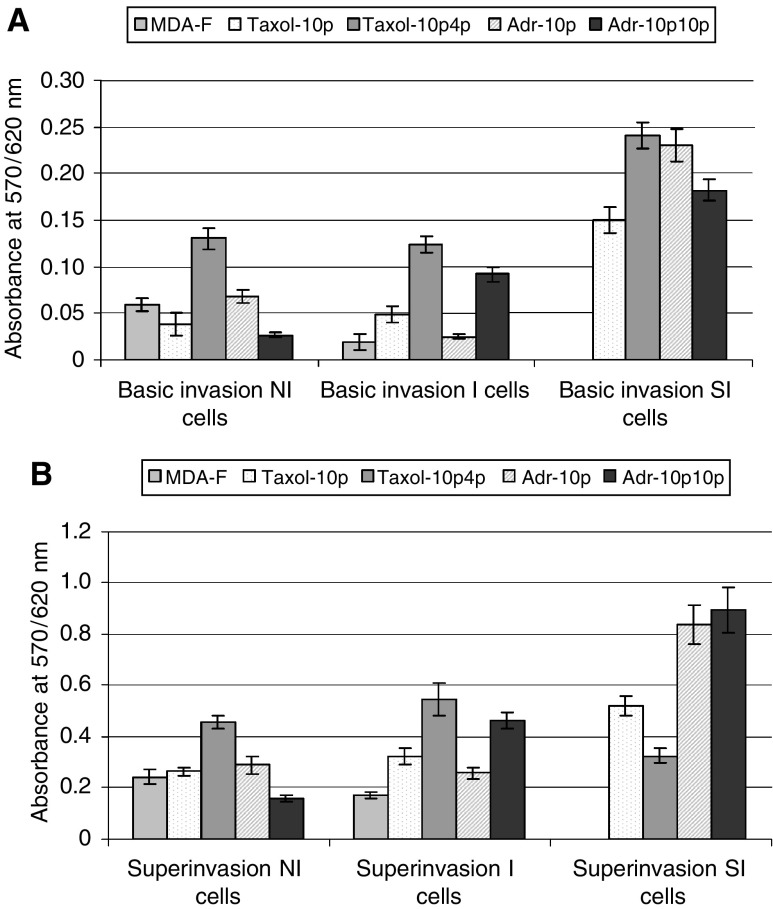
Comparison of levels of (**A**) basic invasion and (**B**) superinvasion by noninvasive (NI), invasive (I) and superinvasive (SI) populations, isolated from MDA-MB-435S-F and its drug-selected variants. Results are expressed as absorbance at 570 nm with a reference wavelength of 620 nm. The data are shown as mean±s.d. (*n*=3).

**Figure 5:**
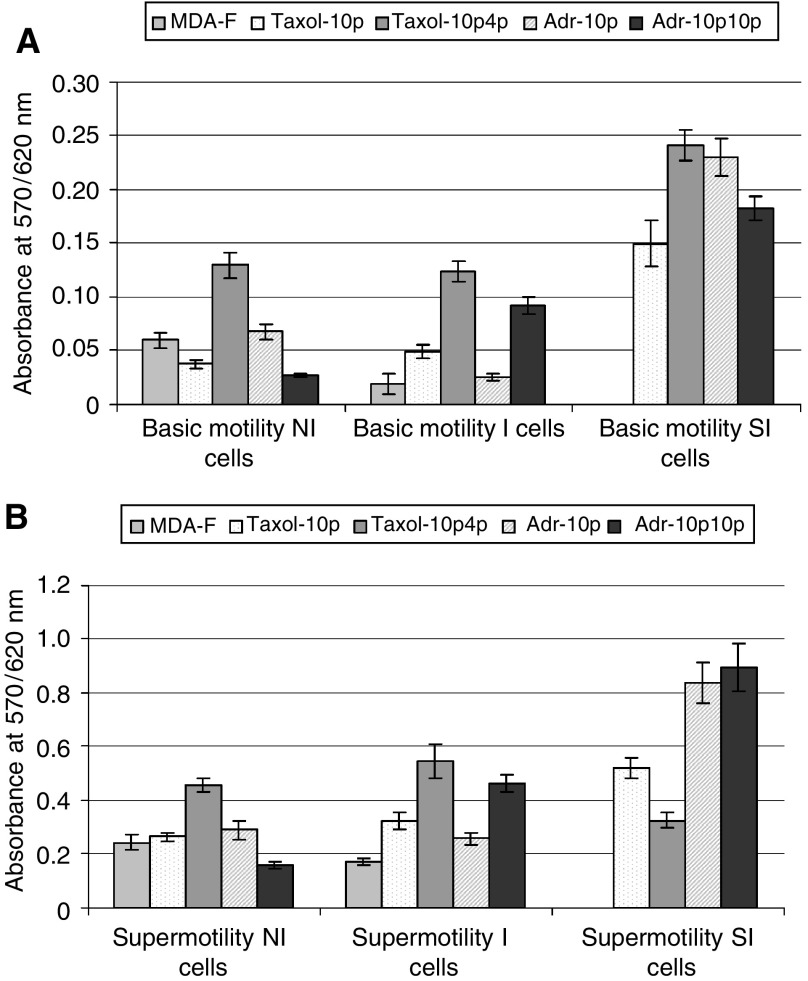
Comparison of levels of (**A**) basic motility and (**B**) supermotility by noninvasive (NI), invasive (I) and superinvasive (SI) populations, isolated from MDA-MB-435S-F and its drug-selected variants. Results are expressed as absorbance at 570 nm with a reference wavelength of 620 nm. The data are shown as mean±s.d. (*n*=3).

Invasion assays were performed to compare the invasiveness of the three populations isolated from MDA-MB-435S-F and its multiple drug-resistant (MDR) variants. Figure 4(a)

represents the levels of cell invasion to the underside of the invasion chamber (basic invasion). The SI cell populations displayed the greatest invasion to this point. Surprisingly, the NI populations displayed invasion similar to the isolated I populations, to the underside of the invasion chamber. Figure 4(b) (a superinvasion assay) represents the relative numbers of cells that relocated to the surface of the 24-well plate after invasion through the invasion chamber. Here, as is the case with motility assays (Figure 5

), invasion was greatest in the SI populations with the exception of MDA-MB-435S-F/Taxol-10p4p. In contrast to Figure 4(a), the I populations displayed greater superinvasion than the NI populations to the surface of the 24-well plate. The SI populations (again with the exception of the MDA-MB-435S-F/Taxol-10p4p SI population) were the most invasive.

### Motility of isolated populations

Motility assays were performed to compare the motility of the three populations isolated from MDA-MB-435S-F and its doxorubicin- and paclitaxel-selected variants. Figure 5(a) represents the levels of cell migration to the underside of the motility chamber (basic motility). Figure 5(b) represents the levels of cell relocation to the surface of the 24-well plate after migration through the motility chamber (supermotility). These figures indicate a general trend for increased motility in the SI populations *vs* NI or I, with the exception of the MDA-MB-435S-F/Taxol-10p4p SI population.

### Adhesion of superinvasive populations to ECM components in comparison to parental populations

Figure 6

**Figure 6:**
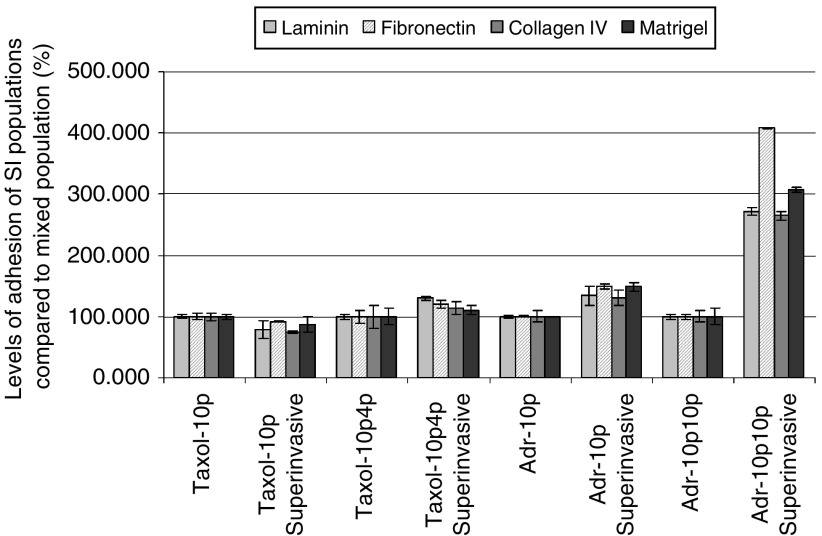
Comparison of adhesiveness of superinvasive populations isolated from MDA-MB-435S-F/Taxol-10p, MDA-MB-435S-F/Taxol-10p4p, MDA-MB-435S-F/Adr-10p and MDA-MB-435S-F/Adr-10p10p to their mixed populations. Adhesion of the superinvasive populations is displayed as a percentage of the adhesion levels of their individual mixed population±s.d. (*n*=3).

shows that the adhesion of the paclitaxel-selected SI populations to ECM proteins was largely unaltered. In contrast, the doxorubicin-selected SI populations displayed increased in adhesion, especially to fibronectin, with increased selection.

### Gelatin zymography

**Figure 7:**
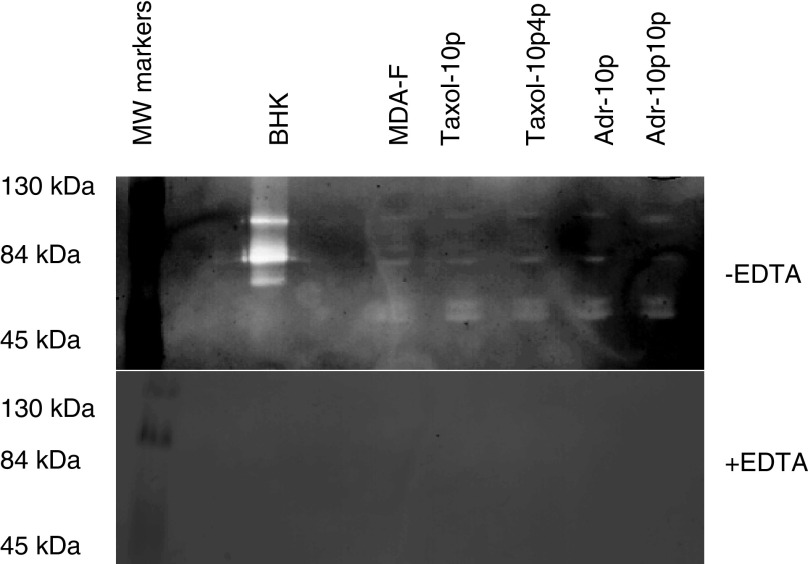
Zymogram of gelatin proteases secreted by MDA-MB-435S-F and its drug-selected variants in the presence or absence of EDTA, with BHK as a positive control for the secretion of pro-MMP2 (72 kDa), MMP-2 (66 kDa), pro-MMP-9 (92 kDa) and MMP9 (86 kDa).

Zymography was also performed as shown in Figure 7

. No alterations in the levels of MMP activity were found. This shows that the superinvasive phenotype is not associated with increased MMP activity. The MDA-MB-435S-F parental cell line and its drug-selected variants display secreted bands corresponding to pro-MMP-2 and pro-MMP-9. There are also double bands expressed at approximately 50–60 kDa. A candidate for this is MMP-1, which runs as a doublet at 52 and 57 kDa in their unglycosylated and glycosylated forms of the proenzyme. Addition of EDTA, a chelating agent which can bind zinc and calcium which are needed for the activation of MMPs, to the substrate buffer eliminates expression of all protease bands, confirming the gelatin degrading proteases secreted by MDA-MB-435S-F parent and variants are most probably members of the matrix metalloproteinase family.

### Mechanisms of increased superinvasiveness

Figure 8

**Figure 8:**
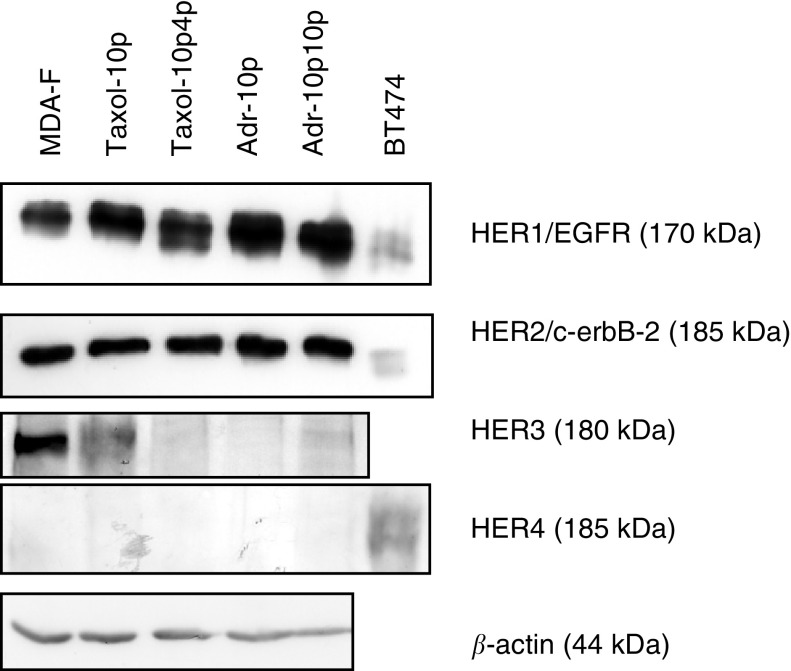
Expression of HER receptor proteins, HER1/EGFR, HER2/c-erbB-2, HER3 and HER4 in MDA-MB-435S-F and its doxorubicin- and paclitaxel-selected variants. *β*-Actin is included as a loading control. This gel is representative of repeat experiments.

shows that HER1/EGFR protein expression was increased with increasing paclitaxel and doxorubicin resistance, while expression of HER2/c-erbB-2 protein remained unchanged. HER3 protein expression decreased with increasing paclitaxel resistance and also with doxorubicin selection. HER4 protein expression was undetectable in any of the MDA-MB-435S-F variants.

**Figure 9:**
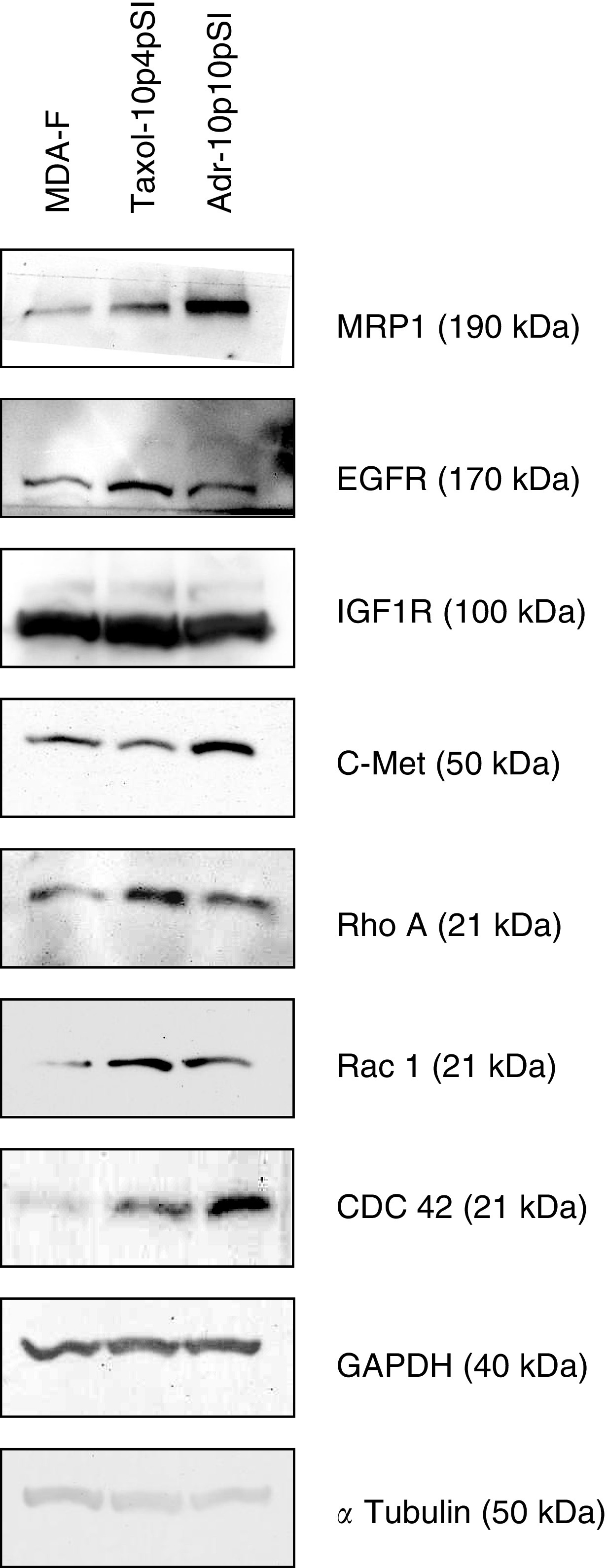
Expression of a range of invasion and motility associated proteins in MDA-MB-435S-F, MDA-MB-435S-F/Taxol-10p4pSI and MDA-MB-435S-F/Adr-10p10pSI. *α*-tubulin and GAPDH are included as a loading controls. This gel is representative of repeat experiments.

In order to investigate changes in protein levels that might be associated with mechanisms for the superinvasive phenotype, Western blots were performed on the parental cell line, MDA-MB-435S-F, and two SI populations MDA-MB-435S-F/Taxol-10p4pSI and MDA-MB-435S-F/Adr-10p10pSI. Proteins included in the investigation were the Rho-like GTPases, cdc42, RhoA and Rac1, the growth factor receptors EGFR/HER1, c-Met, and IGF-1R, and the drug resistance pump MRP1; results are presented in Figure 9

. Levels of Cdc42, Rac1 and RhoA were increased. A 50 kDa portion of c-Met was elevated in MDA-MB-435S-F/Adr-10p10pSI. The IGF-1R receptor remained unchanged, while HER1/EGFR increased. MRP1 was also elevated in the SI populations.

## DISCUSSION

There have been various reports linking development of resistance to chemotherapeutic drugs, such as doxorubicin (Giavazzi *et al*, 1983; Scaddan and Dufresne, 1993), methotrexate (Lucke-Huhle, 1994) and melphalan (Liang *et al*, 2001), with increased invasiveness. The results presented here demonstrate that selection of the human breast cell line MDA-MB-435S-F with doxorubicin and paclitaxel resulted in a more aggressively invasive phenotype. Selection of MDA-MB-435S-F with doxorubicin or paclitaxel had a dramatic effect on the nature of invasion in this cell line; instead of the cells invading to the underside of the invasion chamber and halting at this point (normal invasion), a significant proportion of the cells, which we term superinvasive cells, were observed to detach from the underside of the chamber and reattach to the surface of the 24-well plate. This was not observed with the parental cell line. Enhanced invasiveness following paclitaxel treatment was unexpected, in the context of previous published results (Stearns and Wang, 1992; Terzis *et al*, 1997; Westerlund *et al*, 1997; Liu *et al*, 1998; Sgadari *et al*, 2000). The data presented clearly demonstrate that the superinvasive cells display reduced attachment to ECM proteins coupled with increased motility. It is therefore suggested that these phenomenon contribute to the superinvasive phenotype. The reduction in adhesion was more apparent in the paclitaxel- than in the doxorubicin-selected variants, as was the degree of superinvasiveness. No alterations in the activity of either MMP-2 or MMP-9 were detected. This result was not unexpected as the parental cells already have the ability to travel through the Matrigel and the superinvasive phenotype may essentially involve altered adhesion and motility, coupled with the ability to survive while unattached.

To further investigate the more aggressive superinvasive phenotype observed in the doxorubicin- and paclitaxel-selected variants, three populations (noninvading, NI; invasive, I; superinvasive, SI) were isolated from invasion assays of MDA-MB-435S-F and its selected variants, where available. Analysis of these populations revealed no major differences in their morphology, except that invasive populations tended to clump more. In general, both motility and invasion levels were greatest in the superinvasive populations, followed by the invasive populations, with the noninvading populations being the least motile or invasive; nevertheless, it should be noted that ‘noninvading’ cell population still contained cells capable of invading in subsequent assays. The one exception to this pattern was MDA-MB-435S-F/Taxol-10p4p, with the invasive populations displaying the greatest motility and invasion.

This phenomenon of superinvasion, described for the first time in this paper, may represent an *in vitro* model for a step in the metastasis subsequent to invasion, and hence may lead to a better understanding of the metastatic process.

The Taxol-10p-SI population was less adhesive to the ECM proteins tested than its parental cell line Taxol-10p which in turn was less adhesive than the original cell line MDA-MB-435S-F. The Taxol-10p4p-SI, Adr-10p-SI and Adr-10p10p-SI populations are more adhesive to ECM proteins tested than their individual parental cell lines, which in turn are less adhesive than the original cell line MDA-MB-435S-F.

In order to investigate possible mechanisms of superinvasion, two of the SI populations, MDA-MB-435S-F/Adr-10p10pSI and MDA-MB-435S-F/Taxol-10p4pSI, were compared to the parental cell line MDA-MB-435S-F, for changes in expression at the protein level, of EGFR, the IGF-1 receptor, c-met, the Rho-GTPases, RhoA, Rac1 and Cdc42. Results show Cdc42, Rac1 and RhoA protein increased in the superinvasive populations. Similar patterns have been observed in Ras-transformed fibroblasts and epithelial cells, and may indicate Ras activation in the selected cells (Zondag *et al*, 2000; Sahai *et al*, 2001). RhoA is involved in the generation of contractile force and in moving the body and the tail behind the leading edge. Rac1 is involved in the development of lamellipodia, and Cdc42 is involved in filopodia formation. A 50 kDa truncated form of c-Met was elevated in MDA-MB-435S-F/Adr-10p10pSI. C-met plays a role in inducing invasion and motility. Davies *et al* (2004) demonstrated that by targeting the HGF/SF receptor c-met using a hammerhead ribozyme transgene, they could reduce *in vitro* invasion and migration in prostate cancer cells. IGFR1-R levels did not change in the superinvasive variants, but HER1/EGFR was elevated, as was also seen in MDA-MB-435S-F/Taxol-10p, MDA-MB-435S-F/Taxol-10p4p, MDA-MB-435S-F/Adr-10p and MDA-MB-435S-F/Adr-10p10p, where HER1/EGFR protein was increased and HER3 protein expression was decreased. HER1/EGFR and HER3 have previously been linked to poor prognosis and tumour aggressiveness (Tovey *et al*, 2004; Ueda *et al*, 2004).

Western blot analysis also indicated elevated levels of both P-gp and MRP-1 in the drug-selected variants and SI populations. All the paclitaxel- and doxorubicin-selected variants and SI populations displayed increased doxorubicin efflux, although this is not associated with significant increases in resistance in the doxorubicin-selected variants. By extension this increased efflux may also play a role in paclitaxel resistance in these cell lines. Associations have been shown previously between P-gp overexpression and increased invasiveness (Weinstein *et al*, 1991; Bjornland *et al*, 1998; Kotchetkov *et al*, 2003); however, other studies by Bjornland *et al* (1998) suggests an association rather that a mechanistic link between Pgp expression and invasion.

Sulindac circumvents MRP-1-mediated doxorubicin drug resistance, while verapamil is a modulator of both Pgp- and MRP-1-mediated drug resistance (Duffy *et al*, 1998). Both verapamil and sulindac enhanced doxorubicin toxicity in all cell lines tested. These results suggest that MRP-1 and Pgp both play a role in doxorubicin resistance in these cell lines. Verapamil (but not sulindac) had an effect on paclitaxel resistance in the paclitaxel-selected variants, suggesting a role for Pgp but not MRP-1 (although it must be remembered, in evaluating these results that verapamil and sulindac affect other cellular targets (Etievant *et al*, 1993; Brambilla, 1994; Akashi *et al*, 2000).

Doxorubicin-resistant cell lines that express MRP-1 are normally cross-resistant to doxorubicin, daunorubicin, VP-16, and vincristine, but not to any significant extent to paclitaxel or vinblastine (Haimeur *et al*, 2004). Paclitaxel resistant cell lines that overexpress Pgp are normally cross-resistant to doxorubicin, vincristine, vinblastine, VP-16 and show little or no cross resistance to alkylating agents, platinum drugs and antimetabolites (Sun *et al*, 2004). The paclitaxel-resistant variants discussed here do not display a MDR profile consistent with classic Pgp conferred drug resistance. This resistance profile is more consistent with changes in microtubule expression. These results taken together suggest multiple changes in gene expression in the drug-selected cells.

In summary, the results presented here indicate that exposure of the human breast cell line MDA-MB-435S-F to doxorubicin or paclitaxel can result in an aggressive *in vitro* phenotype not previously described, and which may be a useful model for investigation of a postinvasion step in the metastatic process. While our results suggest that the invasive and superinvasive phenotypes retain their aggressive phenotypes following invasion, we also showed that the noninvasive cells isolated from the wells could subsequently generate invasive and superinvasive populations. This concurs with the complex nature of the invasive phenotype and the apparent ability of the phenotype to revert as evidenced by phenomena such as dormancy of micrometastasis (Naumov *et al*, 2001) and the inefficiency of the metastatic process (Chambers *et al*, 2002).
